# Thermodynamic Characterization of the Interaction of Biofunctionalized Gold Nanoclusters with Serum Albumin Using Two- and Three-Dimensional Methods

**DOI:** 10.3390/ijms242316760

**Published:** 2023-11-25

**Authors:** Ádám Juhász, Gyöngyi Gombár, Egon F. Várkonyi, Marek Wojnicki, Ditta Ungor, Edit Csapó

**Affiliations:** 1 Interdisciplinary Excellence Center, Department of Physical Chemistry and Materials Science, University of Szeged, H-6720 Rerrich B. sqr. 1, 6720 Szeged, Hungaryf.varkonyiegon@chem.u-szeged.hu (E.F.V.); ungord@chem.u-szeged.hu (D.U.); 2 MTA-SZTE Lendület “Momentum” Noble Metal Nanostructures Research Group, University of Szeged, H-6720 Rerrich B. sqr. 1, 6720 Szeged, Hungary; 3 Faculty of Non-Ferrous Metals, AGH University of Science and Technology, Mickiewicza Ave. 30, 30-059 Krakow, Poland; marekw@agh.edu.pl

**Keywords:** gold nanoclusters, bovine serum albumin, surface plasmon resonance, isothermal titration calorimetry

## Abstract

Fluorescent gold nanoclusters have been successfully used as fluorescent markers for imaging of cells and tissues, and their potential role in drug delivery monitoring is coming to the fore. In addition, the development of biosensors using structure-tunable fluorescent nanoclusters is also a prominent research field. In the case of these sensor applications, the typical goal is the selective identification of, e.g., metal ions, small molecules having neuroactive or antioxidant effects, or proteins. During these application-oriented developments, in general, there is not enough time to systematically examine the interaction between nanoclusters and relevant biomolecules/proteins from a thermodynamic viewpoint. In this way, the primary motivation of this article is to carry out a series of tests to partially fill this scientific gap. Besides the well-known fluorescent probes, the mentioned interactions were investigated using such unique measurement methods as surface plasmon resonance (SPR) and isothermal titration calorimetry (ITC). These two-dimensional (at the solid/liquid interface) and three-dimensional (in the bulk phase) measuring techniques provide a unique opportunity for the thermodynamic characterization of the interaction between different gold nanoclusters containing various surface functionalizing ligands and bovine serum albumin (BSA).

## 1. Introduction

In the last decade, the synthesis and characterization of bioligand-stabilized fluorescent noble metal nanostructures have been of interest to researchers [1]. The unique properties of the metal clusters can be tailored through careful engineering of their size, shape, and surface chemistry, allowing for even greater control over their performance and functionality in specific applications. These sub-nanometer-sized clusters show molecular-like spectral performance due to the electronic transitions between HOMO and LUMO energy levels [2]. PTheir unique electronic nature-originated photoluminescent properties are outstanding among nanosized materials and could initiate new opportunities for optical applications. Among the noble-metal-based clusters, the gold nanoclusters (AuNCs) have excellent stability in physiological environmental conditions; moreover, they also show good photostability and low toxicity. These properties make them ideal for use in a wide range of applications, including biomedical devices [1], catalysis [3], and sensor technology [4].

In recent years, the simple, one-pot, “green” syntheses of protein-stabilized AuNCs have been published in numerous works, where it has been proven that the structure of the synthesized nano-object fundamentally depends on the molar ratio of the precursor and the protein. Besides the protein-protected Au clusters, especially small bioligands (e.g., vitamins [5], nucleotides [6], amino- and hydroxamic acids [7,8]), stabilized AuNCs have appeared in the scientific literature as a new class of nanomaterials with great promise in bio-labeling [9], -sensing [10], and -imaging [11,12], as well as targeted cancer therapy applications [13,14].

Although numerous articles and reviews have been published on small bioligand-stabilized fluorescent AuNCs, most of these works focused on their synthesis, physical, and chemical properties, as well as their applications in biosensing and bioimaging utilization. In the case of many types of small-molecule stabilized AuNCs, it can be assumed that they can bind strongly to serum proteins, which affects the pharmacokinetic effect, but the quantitative investigation [15] of the binding process is almost unprecedented. On the other hand, the dominant part of the corresponding publications is summarizing the results of molecular dynamics calculations [16], while there are some publications where the result of the interaction is interpreted as the nature of the protein fluorescence quenching [17,18]. In these research reports, static quenching was identified, which assumes that a non-fluorescent binding complex forms in the interaction of the protein and the cluster as a quencher.

In the present research program, we performed a quantitative characterization of the interaction between previously synthesized and characterized gold nanoclusters and an endogenous protein that is relevant from biological and medical perspectives. In this way, bovine serum albumin (BSA) was chosen as a model molecule of human serum albumin [19], which acts as the main transport protein in the human body. In addition to transporting various endogenous and exogenous substances, it also plays a noteworthy role in maintaining adequate colloid osmotic pressure and buffering the pH of the blood. Besides its enzymatic and antioxidant functions, this macromolecule also acts as a negative acute phase protein, meaning that its concentration alters in cases of inflammation, infection, or cancer diseases. Due to the altered structure of the blood vessel walls surrounding a tumor, the capillaries of the endothelial cells become more permeable, leading to an increase in the amount of serum protein exiting the vessels into the interstitial space, which is known as the enhanced permeability and retention (EPR) effect [20]. The tumor vasculature can be an excellent target for the administration of macromolecular anticancer drugs, which represent the most advantageous drug group in solid tumors [21]. From the point of view of monitoring tumor-selective targeting based on the EPR effect, fluorescent labeling of these drugs with AuNCs as well as the labeling of serum albumin can be particularly beneficial [22].

Considering the above, we investigated the binding affinity of serum albumin with three distinct small molecules of stabilized AuNC. In addition to the conventional spectrofluorimetric measurement, we attempted to characterize the binding process of cluster-protein interaction with previously unutilized methods such as surface plasmon resonance (SPR) spectroscopy and isothermal titration calorimetry (ITC) methods. These techniques provide remarkable opportunities for the thermodynamic characterization of the binding process [23,24]. By combining measurements from both 2D and 3D techniques, we aim to characterize the interaction between the selected gold clusters and proteins using a novel methodology that has not been previously discussed in the literature. Additionally, a combination of these techniques can be used to obtain a comprehensive thermodynamic characterization of the interaction between biofunctionalized AuNCs and proteins. Overall, this thermodynamic information can be used to design novel functionalized AuNCs with optimal properties for specific biomedical applications.

## 2. Results

### 2.1. Optical and Structural Properties of the Biofunctionalized AuNCs

**Figure 1:**
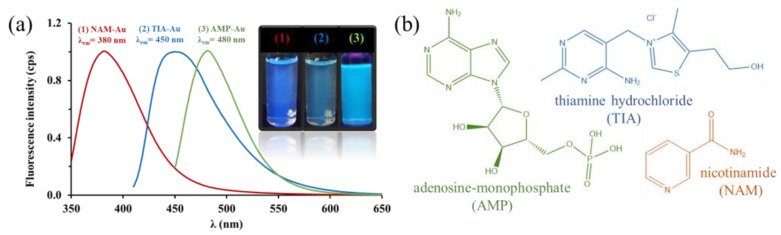
(**a**) Normalized fluorescence spectra of the biofunctionalized gold nanoclusters (1: NAM-AuNCs, 2: TIA-AuNCs, 3: AMP-AuNCs) and photos of the aqueous gold cluster samples illuminated with UV light. (**b**) Chemical structures of the applied ligands for stabilization and biofunctionalization of gold nanoclusters (λ_ex_ = 335, 395, and 335 nm, respectively).

The production and purification of metal nanoclusters stabilized with small molecules were based on the recipes and methods presented in the research group’s relevant publications [5,6,25]. During the synthesis of nicotinamide (NAM) [6], thiamine hydrochloride (TIA) [5], and adenosine monophosphate (AMP) [25] stabilized gold nanoclusters (AuNCs), a cluster dispersion with a volume of 15 cm^3^ was uniformly produced. Then, before using the aqueous colloidal systems for further investigations, the ligand and precursor metal salt remaining in the supernatant were removed by dialysis. During the examination of the spectral differences, we recorded the emission spectra in the wavelength range of 350–650 nm with excitation light of 335 nm (NAM-Au), 395 nm (TIA-Au), and 335 nm (AMP-Au), whose normalized forms are shown in Figure 1a, which is illustrated next to photographs of samples illuminated with UV light. The chemical structures of the applied ligands for stabilization and biofunctionalization of gold nanoclusters are summarized in Figure 1b.

During the structural analysis of the fluorescent products, it was confirmed with X-ray photoelectron spectroscopy (XPS) measurements that the oxidation state of gold is mainly 0, and the coordination of the metal ion to the ligand is confirmed using the results of FT-IR measurements [5,6,25]. In addition to the reproducible and scaled-up reaction conditions used in the present work, the optical properties of the synthesized systems showed good agreement with the previously published results. One of the most suitable analytical methods for the analysis of the composition of nanosized particles is inductively coupled plasma mass spectrometry (ICP-MS), which, thanks to its atto-gram detection limits, can be used to examine the main and minor components of the particles, or even impurities [26,27,28,29]. In this way, the ICP-MS technique has been applied for composition analysis of the purified products of the reproductive syntheses, and it has been established that an average of 95% conversion can be expected during the production of gold nanoclusters [5,6,25].

### 2.2. Surface Plasmon Resonance (SPR) Spectroscopy

**Figure 2:**
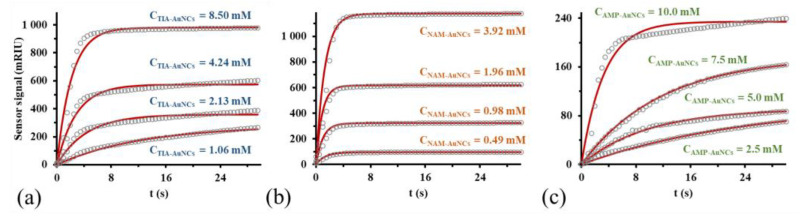
Sensorgrams characterizing the enrichment of gold nanoclusters ((**a**): TIA-AuNCs, (**b**): NAM-AuNCs, and (**c**): AMP-AuNCs) on the BSA functionalized sensor surface (gray circles) and model curves fitted based on the rate equation (Equation (1)) (red solid lines). The *y*-axes of the figures both represent the change in the sensor signal.

The implementation of SPR measurements can typically be divided into two steps. The first is the preparation of the functionalized sensor surface, followed by the examination of the interaction between the receptor bound to the sensor surface and the ligand(s). By closing the reference channel of the microfluidic system of the device, the aqueous solution of BSA with a concentration of 30 µM was delivered into the primary channel at a flow rate of 50 µL min^−1^, while the thermostat and controller integrated in the device ensured a constant temperature of 25 ± 0.2 °C in the vicinity of the sensor surface. In the second phase, corresponding solutions of the known concentration of the ligand are flowed over the sensor surface, while the temporal change of the sensor signal is continuously recorded, which is displayed with gray circles in the sensorgrams of Figure 2.

If we assume that the surface complex formed through the connection of BSA and NCs is the result of a reversible bimolecular reaction, the integrated rate equation describing the increase in the concentration of the binding complex (Equation (1)) can be written in the following form [30,31]:

(1) $$\left[BSA-AuNCs\right]={\left[AuNCs\right]}_{0}\left(1-e^{-k_{obs}t}\right)$$

where [BSA − AuNCs] is the concentration of the binding complex on the sensor surface, [AuNCs]_0_ is the concentration of the used cluster dispersions, and *k_obs_* is the apparent rate constant of the binding process. Considering that the temporal change of [BSA − AuNCs] is proportional to the temporal change of the sensor signal, while the magnitude of [AuNCs]_0_ is proportional to the maximum surface complex concentration (maximum signal shift) achievable under the given conditions, concentration dimensioned terms of Equation (1) can be replaced with the sensor signal [32,33]. With a given analyte concentration, the *k_obs_* value assigned to the given concentration can be derived as a fitting parameter based on the recorded sensorgram. Since *k_obs_* is a linear function (*k_obs_* = *k_a_*[AuNCs]_0_ + *k_d_*) of the concentration of the ligand (biofuntionalized AuNCs), by plotting the *k_obs_* values as a function of the ligand concentration, the real rate constants (*k_a_* and *k_d_*) can be calculated using linear regression (presented in Figure S1). The quotient of the real rate constants provides the equilibrium constant (*K_A_*) for the bonding process, which was found to be 5468 ± 73 M^−1^ for the TIA-AuNCs/BSA system. Oppositely, the value of *K_A_* for the NAM-AuNCs/BSA and AMP-AuNCs/BSA systems was an order of magnitude lower, namely 143 ± 30 M^−1^ and 342 ± 47 M^−1^.

### 2.3. Measurement of Ligand Binding Energetics using Isothermal Titration Calorimetry (ITC)

**Figure 3:**
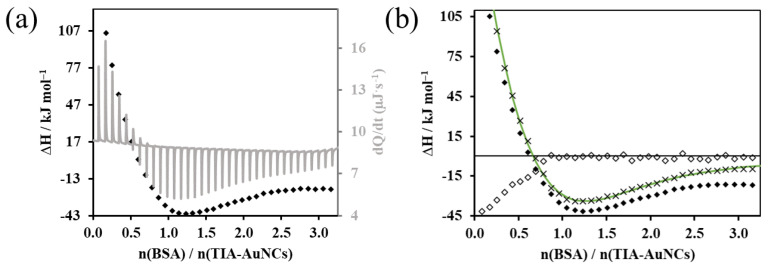
(**a**) Calorimetric curve (gray line) and enthalpogram (♦) recorded during the ITC examination of the BSA/TIA-AuNCs system. (**b**) Experimental- (♦), background- (◊), and dilution-corrected (˟) enthalpogram fitted based on the model assuming two binding sites (green line).

During the calorimetric tests, the 1.4 mL volume and 0.1 mM concentration dispersions of the gold nanoclusters produced during the reproductive syntheses were added to the 1.1 mM concentration BSA solution in 35 steps at 25 °C with constant stirring. During each dosing step, the protein solution with a volume of 8 µL was injected in 16 s, followed by the next injection after 400 s. The thermal information (dQ/dt) collected during the measurements is displayed by the differential calorimetric curve registered as a function of time (indicated with the gray solid line in Figure 3a). The enthalpograms represent the enthalpy change values related to the amount of protein added to the system as a function of the amount of substance ratio (n_protein_/n_cluster_) that can be assigned to each dosing step. The heat effect resulting from protein dilution can be corrected with background titration [34]. This dilution/background enthalpogram (displayed with the symbol ◊ in Figure 3b) is subtracted from the data of the experimental enthalpogram to give the corrected enthalpogram, which contains only the thermal information resulting from the interaction of the cluster and the protein. The binding model, assuming two binding sites, was suitable for describing the experimental data [35,36]. In this way, the thermodynamic parameters provided by the theoretical enthalpogram indicated with the green line in Figure 3b can be accepted as characteristic values of the binding process. Table S1 summarizes the value and standard deviation of the equilibrium constant (*Ka*), the free enthalpy change (Δ*G*), and the enthalpy change (Δ*H*) of the binding of NAM-AuNCs to the protein, as well as the binding stoichiometry (*N*) of each binding site.

Considering the standard deviation of the data summarized in Table S1, it can be concluded that only the data relating to the first (essentially 1:1 stoichiometry) binding site can be accepted as the values describing the binding. Namely, the values of the thermodynamic parameters calculated for the primary binding site are as follows: *K_a_* = 4.17∙10^4^ ± 9.2∙10^3^ M^−1^; Δ*G* = −26.36 ± 0.55 kJ∙mol^−1^; Δ*H* = −230 ± 181 kJ∙mol^−1^; and *N* = 1.19 ± 0.14. Based on these, the incorporation of the cluster is the result of an exothermic process occurring spontaneously at the tested temperature. If we consider the negative ΔH value and the small but also negative ΔS value (−682 ± 4 J∙mol^−1^∙K^−1^), we can also conclude that secondary forces such as H-bonding and electrostatic interaction play a role in the outstanding affinity of TIA-AuNCs to the protein [37,38].

Figures S2 and S3 show the analog ITC results of the binding of NAM-AuNCs and AMP-AuNCs to the protein, along with the calculated enthalpogram. In the case of NAM-AuNCs, the theoretical enthalpogram, which also assumes two binding sites, was able to well describe the evolution of the experimental data. In the case of NAM-AuNCs, the theoretical enthalpogram, which also assumes two binding sites, was able to well describe the evolution of the experimental data. The evaluated quantities for the primary binding site are as follows: *K_a_* = 4.66∙10^5^ ± 6.9∙10^5^ M^−1^; Δ*G* = −32.34 ± 3.67 kJ∙mol^−1^; Δ*H* = −5.58 ± 8.98 kJ∙mol^−1^; and *N* = 1.09 ± 0.31 (detailed data summarized in Table S2).

The enthalpograms of the AMP-AuNCs/macromolecule interaction are presented in Figure S3a. The calculated enthalpogram of Figure S3b provides the following quantities for the primary binding site: *K_a_* = 1.64∙10^5^ ± 1.6∙10^4^ M^−1^; Δ*G* = −29.74 ± 0.24 kJ∙mol^−1^; Δ*H* = −12.39 ± 5.36 kJ∙mol^−1^; and *N* = 0.60 ± 0.37 (detailed data shortened in Table S3).

Due to the uncertainty arising from the large standard deviation of the determined parameters, the binding of the NAM-AuNCs and AMP-AuNCs to the protein cannot be clearly verified based on the ITC analysis. Considering this uncertainty, in the case of ITC results, we refrain from further and more detailed thermodynamic analysis of the interaction between these nanoclusters and the protein.

### 2.4. Photoluminescence (PL) Spectroscopy Investigation of Albumin Fluorescence Quenching

**Figure 4:**
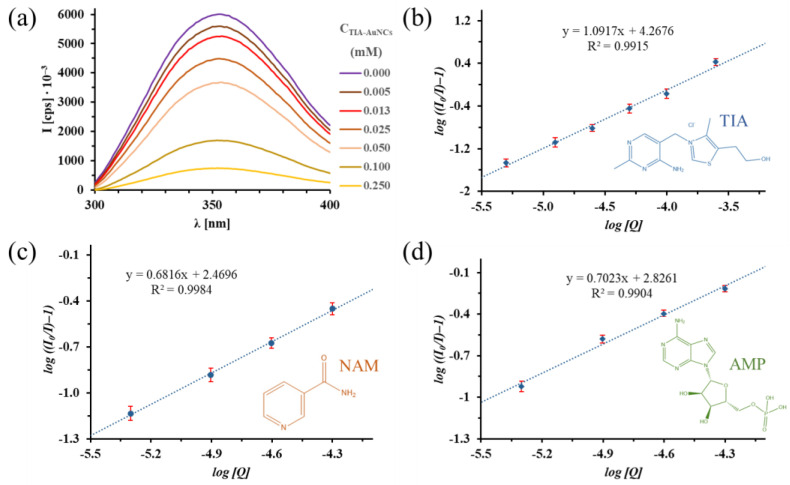
(**a**) Fluorescence emission spectra of the aqueous BSA solution and the protein/cluster mixtures after addition of 0–1 mM TIA-AuNCs with constant albumin concentration (c_BSA_ = 0.5 mM; T = 25 °C; and λ_ex_ = 280 nm) and the Stern–Volmer representation of the experienced quenching of BSA fluorescent emission at 350 nm with gold nanoclusters ((**b**): TIA-AuNCs, (**c**): NAM-AuNCs, and (**d**): AMP-AuNCs).

To further investigate the functionalized AuNCs-BSA binding process, PL measurements were carried out. The fluorescence of Trp amino acids in the protein chain depends on the chemical environment. In this way, the conformation change of the protein has a significant effect on the extent of the intensity and the energy distribution of the emitted photons. As it can be seen in Figure 4a, the intensity of the Trp emission decreases with an increase in the TIA-AuNCs concentration. The binding constant and the stoichiometry of the protein–cluster interaction, studied with the intrinsic fluorescence quenching of BSA via TIA-AuNCS, reflect the strength of the interaction. These can be determined using the double logarithmic Scatchard equation (Equation (2)) [39,40,41]:

(2) $$log\left(\frac{I_{0}}{I}-1\right)=logK_{a}+N\cdot log\left[Q\right]$$

where *I*_0_ and *I* are the fluorescence intensities of the protein in the absence and presence of the AuNCs, *K_a_* is the binding (association) constant, *N* is the number of binding sites of albumin towards the ligand, and [*Q*] is the concentration of the quencher (in this present case, the concentration of the AuNCs).

With the linear regression of the experimental data (Figure 4b), the calculated *K_a_* value was found to be 1.85∙10^4^ ± 4.01∙10^3^ M^−1^, which indicates a strong interaction between the TIA-AuNCs and albumin [42,43]. The determined *N* is 1.01 ± 0.11 and the calculated Gibbs energy change of the interaction is −24.35 ± 0.12 kJ·mol^−1^, which confirms a spontaneous binding process.

During the further PL studies of the other two AuNCS, the same methodology was used. The BSA concentration was fixed at 5.0 µM, and the added cluster amount was altered, while the concentration of the quencher increased from 0 to 0.02 mM. Fluorescence emission spectra of the aqueous albumin solution and the protein/cluster mixtures (Figure S4a) after addition of AuNCs and the Stern–Volmer representations of the BSA/NAM-AuNCs system are represented in Figure 4c. The analougous experimental spectral data sets (Figure S4b) and the evaluation of the fluorescence quenching for the BSA/AMP-AuNCs system are shown in Figure 4d. For the BSA/NAM-AuNCs system, the Stern–Volmer-method-evaluated (Figure 4c) values were found to be as follows: *K_a_* = 295 ± 0.2 M^−1^, *N* = 0.68 ± 0.03, and ΔG = −14.09 ± 0.11 kJ·mol^−1^.

For the BSA/AMP-AuNCs system, the linear-regression-based (Figure 4d) values of the quenching process were found to be as follows: *K_a_* = 670 ± 3 M^−1^, *N* = 0.70 ± 0.09 and ΔG = −16.12 ± 0.14 kJ·mol^−1^. These results show a significantly weaker interaction between NAM- and AMP-AuNCs and albumin, in agreement with the results from the previously presented SPR and ITC measurement techniques.

## 3. Discussion

**Figure 5:**
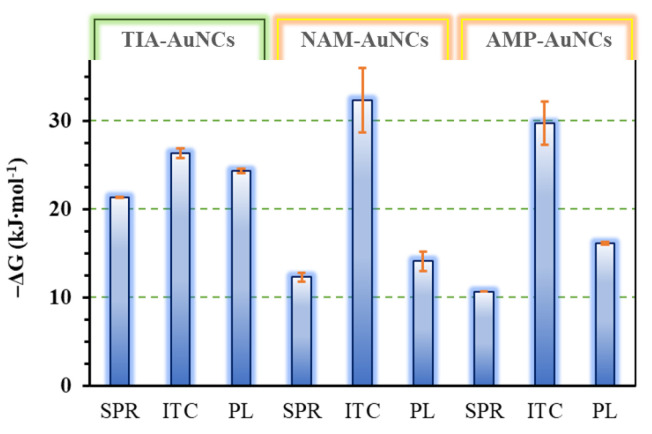
The opposite of the experimental Gibbs free energy change (−Δ*G*) of the binding interaction between AuNCs and BSA from SPR, ITC, and PL spectroscopy measurement-based evaluation methods.

A possible binding interaction between AuNCs and BSA was investigated using SPR, ITC, and PL spectroscopy methods. Figure 5 summarizes the alteration of the Gibbs free energy changes of the investigated interaction partners, while the whole thermodynamic parameter set that was derived experimentally via the application of the listed measurement techniques is collected in Table S4. Knowledge of these characteristic parameters gives the opportunity for a quantitative description of the receptor–ligand-type interaction between the gold nanoclusters and albumin. Moreover, these thermodynamic characteristics can help to discover the possible binding mechanism and contribute to the recognition of those cluster types that can be effectively used as sensors of biological systems or fluorescent markers for nanosized drug delivery systems [44]. Looking at the development of the free energy values summarized in Figure 5, it can be concluded that the applied measurement techniques only provide coherent results regarding the binding to the protein in the case of the vitamin B1 (TIA) stabilized gold nanocluster. In the case of the TIA-AuNCs, the SPR, ITC, and Pl measurements all confirm that the cluster forms a strong bond with the protein during a spontaneous process that can be described with a 1:1 stoichiometry.

Complementing the data delivered using independent measurement methods with the detailed thermodynamic information provided through the ITC test, the following considerations help in a more detailed characterization of the investigated interaction. A combination of numerous forces may be involved in the interaction between the proteins and their ligands, namely the hydrogen bonds, electrostatic, hydrophobic, and van der Waals forces [45,46]. The sign and magnitude of the Gibbs free energy, enthalpy, and entropy changes of the binding process can be correlated with the types of interactions that may occur in protein association processes [47].

Based on the outcomes of the independent techniques (listed in Table S4), the interaction of TIA-AuNCS with the protein is a spontaneous process (Δ*G* < 0), and based on the ITC results, the entropic and enthalpic terms show that the binding complex formation is an exothermic process, while the reaction is enthalpically driven at the investigated temperature. Additionally, considering that Δ*H* < 0 and Δ*S* < 0, one can deduce that the hydrogen bonding and van der Waals forces are the main driving forces of the binding process, as other publications suggest. These results agree with the results of previous studies in the case of similar proteins, Au-NCs, or other particles [33,48,49].

## 4. Materials and Methods

### 4.1. Chemicals

The reproductive synthesis of fluorescent gold nanoclusters (TIA-AuNCs: [5]; NAM-AuNCs: [6]; and AMP-AuNCs: [25]) was carried out according to the recipes presented in detail in the relevant publications, using the below-listed chemicals. All chemicals were of analytical grade and were used without further purification. Nicotinamide (NAM; C_6_H_6_N_2_O; ≥99.5% (HPLC)), adenosine 5′-monophosphate disodium salt (C_10_H_12_N_5_Na_2_O_7_P, 99.0%), thiamine hydrochloride (Vitamin B_1_; C_12_H_17_ClN_4_OS·HCl; ≥99.9%), gold(III) chloride acid trihydrate (HAuCl_4_ × 3H_2_O, 99.9%), and lyophilized powder of bovine serum albumin (BSA ≥ 98.0%, M_w_ = 66 kDa) were obtained from Sigma-Aldrich Hungary Ltd. (Budapest, Hungary). In all cases, the stock solutions were freshly prepared using Milli-Q ultrapure water (18.2 MΩ·cm at 25 °C).

### 4.2. Methods

#### 4.2.1. Surface Plasmon Resonance (SPR) Spectroscopy

To identify AuNC interactions with BSA in vitro, surface plasmon resonance (SPR) experiments [50,51] were performed using a dual-channel Reichert SPR spectrometer (SR7500DC; Reichert, Depew, NY, USA). 50 nm gold-coated SPR gold chips, supplied by Reichert Technologies, were used for studying the binding reaction on SPR. Prior to their modification, the chips were cleaned using piranha solution (3:1 H_2_SO_4_:H_2_O_2_) for 20 s, rinsed thoroughly with MilliQ water, and finally dried with nitrogen gas. After the functionalization of the sensor surface, samples of different concentrations of the selected AuNCs were applied to interact with the immobilized BSA surface while continuously recording the changes in the sensor signal over time. The association time was 60 s for 30 μL/min.

#### 4.2.2. Isothermal Titration Calorimetry (ITC)

Isothermal Titration Calorimetry (ITC) measurements were performed with a VP-ITC microcalorimeter from MicroCal Inc. (Northampton, MA, USA). During the calorimetric tests, 8 µL per volume unit of the BSA solution was injected in 35 steps into the 1.4 mL volume dispersions of the gold nanoclusters with constant stirring. In each dosing step, the protein solution with a volume of 8 µL was injected in 16 s, followed by the next injection after 400 s. For the extraction of the dilution effect from the registered enthalpograms, the corresponding reference blank trials were also performed under the same conditions [52,53]. Namely, the titration of protein solution in MilliQ water was carried out with 8 µL per volume unit of the BSA solution in 35 steps. To avoid the presence of bubbles, all samples were degassed for 10 min shortly before starting the measurements. The sample cell was constantly stirred at a rate of 240 rpm, and the measurements were performed at 25 °C.

#### 4.2.3. Photoluminescence (PL) Spectroscopy

Fluorescence measurements were carried out in a Horiba Jobin Yvon FloroMAx-4 type spectrofluorometer using a flow-through quartz cuvette of 1 cm path length connected to a thermostat. Fluorescence quenching studies have been performed using a BSA solution of 0.5 mmol·L^−1^ concentration, and the amount of the AuNCs ranged from a 0.0 to 1.0 mmol·L^−1^ concentration value at 25 ± 1 °C, controlled with a Peltier thermostat. The samples were allowed to equilibrate for 15 min prior to measurement. The fluorescence spectra of the samples were recorded in the wavelength range of 305–425 nm upon excitation at 395 nm with a 2 nm slit.

## 5. Conclusions

The interaction between AuNCs and BSA was investigated using surface plasmon resonance (SPR) spectroscopy, isothermal titration calorimetry (ITC), and fluorescent spectroscopy. Processing and evaluation of the SPR platform recorded sensorgrams provided the determination of the apparent rate constant of the nanocluster binding. Through the concentration dependence of this apparent parameter, the real rate constants have been calculated, and their quotient has provided the value of the equilibrium constant (*K_a_*). Modelling the corrected experimental enthalpograms of ITC measurements using different binding models resulted in the corresponding thermodynamic parameters (*K_a_*, Δ*G*, and Δ*H*) of the binding process, as well as the value of the binding stoichiometry of the individual binding sites. The results of the spectrofluorimetric analysis of the assumed binding process were analyzed with the Scatchard representation, and the value of the association constant of the binding process and the number of protein binding sites were successfully calculated in the case of TIA-AuNCs. Based on the comparison of the results of the various measurement techniques, the binding of the B1-stabilized gold nanocluster to BSA is the result of an exothermic, spontaneously occurring hydrogen bond, and electrostatic interaction-type secondary forces play a major role. The cluster that is located at one of the two Sudlow’s sites of the protein with a 1:1 stoichiometry modifies the structure of the albumin and prevents the other binding site from fulfilling its role with the same affinity.

## Data Availability

The data sets used and evaluated during the presented study are available from Ádám Juhász (juhaszad@chem.u-szeged.hu) upon reasonable request.

## Supplementary Materials

The supporting information can be downloaded at https://www.mdpi.com/article/10.3390/ijms242316760/s1.
