# Supplementary material for: Thermodynamic Characterization of the Interaction of Biofunctionalized Gold Nanoclusters with Serum Albumin Using Two- and Three-Dimensional Methods
Source: Int J Mol Sci. 2023 Nov 25;24(23):16760. doi: 10.3390/ijms242316760 (PMC10706308; doi:10.3390/ijms242316760)
Supplement: Supplementary file 1 [file ijms-24-16760-s001.zip › ijms-2729105-supplementary.pdf]

International Journal of *Molecular Sciences*

MDPI

Supplementary Materials

Thermodynamic Characterization of the Interaction of Biofunctionalized Gold Nanoclusters with Serum Albumin Using Two- and Three-Dimensional Methods

Ádám Juhász [1,2,*], Gyöngyi Gombár [1,2], Egon F. Várkonyi [1], Marek Wojnicki [3], Ditta Ungor [1] and Edit Csapó [1,2,*]

1 Interdisciplinary Excellence Center, Department of Physical Chemistry and Materials Science, University of Szeged, H-6720 Rerrich B. sqr. 1, 6720 Szeged, Hungary; gygombar@gmail.com (G.G.); f.varkonyiegon@chem.u-szeged.hu (E.F.V.); ungord@chem.u-szeged.hu (D.U.)

2 MTA-SZTE Lendület "Momentum" Noble Metal Nanostructures Research Group, University of Szeged, H-6720 Rerrich B. sqr. 1, 6720 Szeged, Hungary

3 Faculty of Non-Ferrous Metals, AGH University of Science and Technology, Mickiewicza Ave. 30, 30-059 Krakow, Poland; marekw@agh.edu.pl

* Correspondence: juhaszad@chem.u-szeged.hu (Á.J.); juhaszne@chem.u-szeged.hu (E.C.)

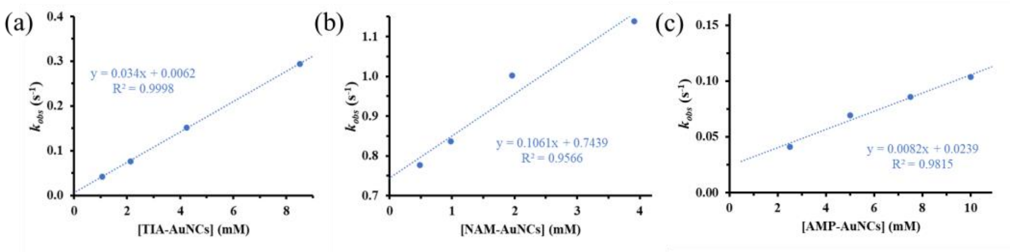

Figure S1. Determination of the real rate constants (k_a and k_d) of the binding process to BSA based on the concentration dependence of the apparent rate constant (k_{obs}) for TIA (**a**), NAM (**b**) and AMP (**c**) stabilized AuNCs.

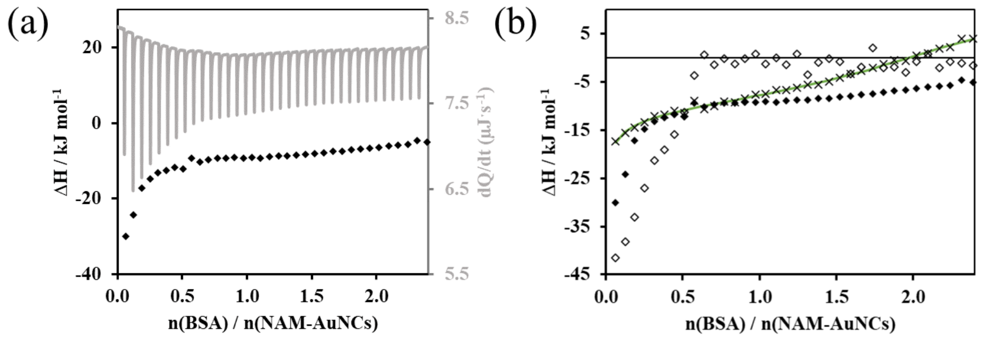

Figure S2. (**a**) Calorimetric curve (gray line) and enthalpogram (♦) recorded during the ITC examination of the BSA/NAM-AuNCs system; (**b**) Experimental- (♦), background- (◊) and dilution corrected- (×) enthalpogram fitted based on the model assuming two binding sites (green line).

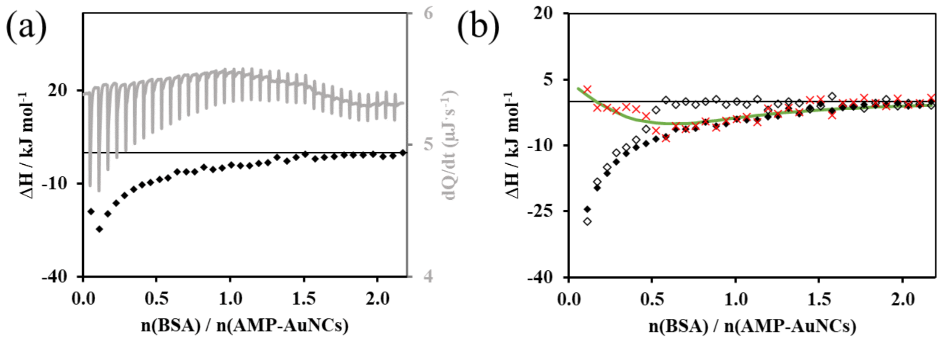

Figure S3. (**a**) Calorimetric curve (gray line) and enthalpogram (♦) recorded during the ITC examination of the BSA/NAM-AuNCs system; (**b**) Experimental- (♦), background- (◊) and dilution corrected- (×) enthalpogram fitted based on the model assuming two binding sites (green line).

Table S1. The value and standard deviation of the thermodynamic parameters determined based on the calorimetric (ITC) analysis of the interaction between TIA-AuNCs and BSA.

$K_{a,1}$ (M^{-1})	$K_{a,2}$ (M^{-1})
$4.17 \cdot 10^4 \pm 9.20 \cdot 10^3$	$1.04 \cdot 10^5 \pm 7.60 \cdot 10^4$
ΔG_1 (kJ·mol^{-1})	ΔG_2 (kJ·mol^{-1})
-26.36 ± 0.55	-28.62 ± 1.81
ΔH_1 (kJ·mol^{-1})	ΔH_2 (kJ·mol^{-1})
-230 ± 181	1400 ± 3600
N_1	N_2
1.19 ± 0.14	0.29 ± 0.18

Table S2. The value and standard deviation of the thermodynamic parameters determined based on the calorimetric (ITC) analysis of the interaction between NAM-AuNCs and BSA.

$K_{a,1}$ (M^{-1})	$K_{a,2}$ (M^{-1})
$4.66 \cdot 10^5 \pm 6.90 \cdot 10^5$	$2.12 \cdot 10^6 \pm 3.80 \cdot 10^6$
ΔG_1 (kJ·mol^{-1})	ΔG_2 (kJ·mol^{-1})
-32.34 ± 3.67	-36.09 ± 4.44
ΔH_1 (kJ·mol^{-1})	ΔH_2 (kJ·mol^{-1})
-5.58 ± 8.98	-27.71 ± 23.28
N_1	N_2
1.09 ± 0.31	0.44 ± 0.25

Table S3. The value and standard deviation of the thermodynamic parameters determined based on the calorimetric (ITC) analysis of the interaction between AMP-AuNCs and BSA.

$K_{a,1}$ (M^{-1})	$K_{a,2}$ (M^{-1})
$1.64 \cdot 10^5 \pm 1.60 \cdot 10^5$	$1.10 \cdot 10^6 \pm 2.80 \cdot 10^6$
ΔG_1 (kJ·mol^{-1})	ΔG_2 (kJ·mol^{-1})
-29.75 ± 2.42	-34.47 ± 6.31
ΔH_1 (kJ·mol^{-1})	ΔH_2 (kJ·mol^{-1})
-12.39 ± 5.36	5.821 ± 8.66
N_1	N_2
0.60 ± 0.37	0.37 ± 0.16

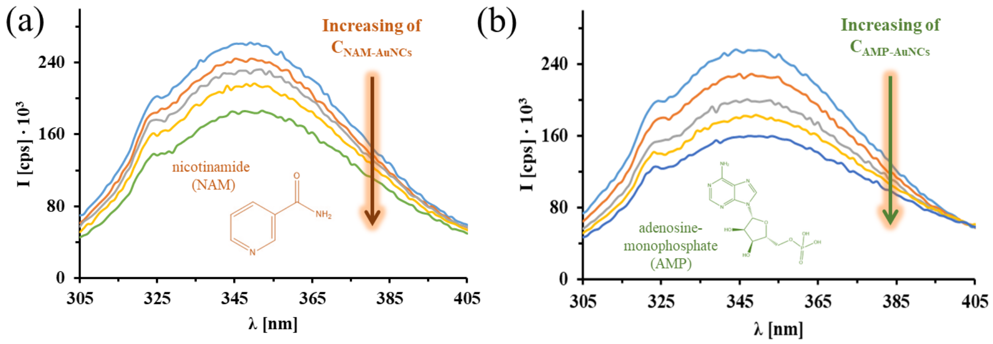

Figure S4. (**a**) Fluorescence emission spectra of the aqueous BSA solution and the protein/cluster mixtures after addition of 0–1 mM NAM-AuNCs with constant albumin concentration (c_{BSA} = 5.0 µM; T = 25 °C; λ_{ex} = 280 nm) (**b**) Fluorescence emission spectra of the aqueous BSA solution and the protein/cluster mixtures after addition of 0–1 mM AMP-AuNCs with constant albumin concentration (c_{BSA} = 5.0 µM; T = 25 °C; λ_{ex} = 280 nm)

Table S4. The value and standard deviation of the thermodynamic parameters of the examined gold nanocluster-protein interactions, evaluated from SPR, ITC and PL measurement techniques.

		K_a (M^{-1})	ΔG (kJ·mol^{-1})	ΔH (kJ·mol^{-1})	N	ΔS (kJ·mol^{-1})
SPR	TIA	5468 ± 73	-21.32 ± 0.03			
	NAM	143 ± 30	-12.29 ± 0.53			
	AMP	294 ± 90	-14.08 ± 0.76			
ITC	TIA	$4.17 \cdot 10^4 \pm 9.20 \cdot 10^3$	-26.36 ± 0.55	-230 ± 182	1.19 ± 0.14	-682 ± 4
	NAM	$4.66 \cdot 10^5 \pm 6.90 \cdot 10^5$	-32.34 ± 3.67	-5.28 ± 8.95	1.19 ± 0.14	91 ± 13
	AMP	$1.64 \cdot 10^5 \pm 1.60 \cdot 10^5$	-34.47 ± 2.42	-12.39 ± 5.36	1.19 ± 0.14	58 ± 19
PL	TIA	$1.85 \cdot 10^4 \pm 1.80 \cdot 10^3$	-24.35 ± 0.24		1.19 ± 0.14	
	NAM	295 ± 1	-14.09 ± 0		1.19 ± 0.14	
	AMP	640 ± 3	-16.12 ± 0		1.19 ± 0.14	
